# Flavored Vitamin–Mineral Supplementation via Water Improves the Performance of Newly Weaned Piglets

**DOI:** 10.1111/asj.70166

**Published:** 2026-02-21

**Authors:** Pedro Henrique Inácio Gomes, Márvio Lobão Teixeira de Abreu, Claudia Cassimira Silva, Adsos Passos, Mauricio Prata, Mara da Silveira Benfato, Diego Mena Canata, Alejandra Gutierezz Riaño, Afonso Luna Miranda, Isabella Santos Correa, Beatriz Rodrigues Siqueira, Bruno Alexander Nunes Silva

**Affiliations:** ^1^ Department of Animal Sciences Universidade Federal de Lavras Lavras Minas Gerais Brazil; ^2^ Institute of Agricultural Sciences Universidade Federal de Minas Gerais (UFMG) Montes Claros Minas Gerais Brazil; ^3^ Animal Nutrition & Health dsm‐firmenich São Paulo Brazil; ^4^ Institute of Biosciences/IBIO Universidade Federal do Rio Grande do Sul (UFRGS) Porto Alegre Rio Grande do Sul Brazil

**Keywords:** feeding behavior, nursery, oxidative stress, pigs, weaning

## Abstract

The aim was to evaluate the supplementation of weaned piglets using a water‐soluble flavored vitamin–mineral mixture. Ninety weaned piglets were distributed in a randomized block design with three treatments: control (CON), no supplement during nursery; W5D, with supplementation for 5 days after weaning and another 5 days before the end of Phase 3 and beginning of Phase 4; and W10D, with supplementation during the first 10 days after weaning. The W10D showed higher daily weight gain (*p* = 0.047) in Phase 3 and higher water expenditure during Phase 3 and Phase 4 (*p* < 0.05). The W5D and W10D reduced superoxide dismutase activity in erythrocytes and malondialdehyde concentration in plasma, indicating a positive effect on redox status. Behavioral assessment showed that W10D piglets spent more time on the feeder in the first 24 h after weaning compared with other groups (*p* = 0.045). Incidence of diarrhea was lower in the W5D and W10D treatments during the last phase (*p* = 0.002) and lower for the W10D treatment throughout the entire experimental period (*p* < 0.001). In conclusion, supplementation of a flavored vitamin–mineral mixture via water during the first 10 days postweaning improves the performance of piglets, with beneficial effects on redox status, behavior, and a reduction in the incidence of diarrhea.

## 1 Introduction

Weaning is one of the main and most critical managements in swine production, characterized by the high intensity of stress caused to piglets due to the separation from the dam, as well as changes in the environment, changes in the feeding regime, and changes in the social circle, all of which occur abruptly (Moeser et al. 2017). This stress can trigger different consequences that impact the piglets' feeding pattern, gut integrity, metabolism, and immune status (Colson et al. 2012; Degroote et al. 2020), all in all harmful to health and deleterious to performance. Social stress is favored by comingling piglets of different litters and by the animals' immaturity to tolerate weaning management. Thus, competition and the occurrence of fights to reestablish a new hierarchy in the pen also have a negative impact on the piglets' productive performance (Camerlink et al. 2021). This resocialization process is the main cause of fighting among newly weaned piglets (Worobec et al. 1999), resulting in an increase in cortisol concentration and negatively influencing individual performance (Salazar et al. 2018).

In addition, stress will also impact feeding and drinking behaviors, limiting water consumption (Brooks et al. 1984), which is another reason for performance loss in the postweaning period (da Silva et al. 2020). Other studies have shown that there is a relationship between water consumption and feed efficiency and growth in nursery piglets (Nienaber and Leroy Hahn 1984), which may also be related to gastrointestinal dysfunction. There is still little discussion and little information on the impact of weaning on water intake and consequently on piglet health. Water intake limitation intensifies stress after weaning by increasing serum cortisol concentration, indicating that water deprivation is a more potent psychological stressor when compared with feed deprivation, suggesting a subsequent impact on the performance of nursery piglets (Horn et al. 2014).

Oxidative stress occurs due to an imbalance in the redox status, i.e., an imbalance between the production of reactive oxygen species (ROS) and their neutralization by the body's antioxidant system. In addition, oxidative stress is responsible for damaging the integrity of the intestinal barrier, potentializing immune challenges (John et al. 2011; Cao et al. 2018). The effects are detrimental to piglets, as the process of cellular oxidation is rapidly intensified soon after weaning and its effects remain for weeks (Novais et al. 2020). As a result of the damage caused by the imbalance of the redox status, there is a response from the immune system, increasing the release of proinflammatory cytokines, disturbing the immune system, and developing a process of intestinal inflammation (Lykkesfeldt and Svendsen 2007; Moeser et al. 2017). Therefore, nutritional and management strategies should be adopted to optimize water intake and reduce oxidative and psychological stress in weaned piglets.

Vitamins are important biological components for piglets in the postweaning period and can be used as functional nutrients in the intestinal development (Li et al. 2020), modulation of the immune response (Cantorna et al. 2019), inflammation control, and neutralization of free radicals in the intestine (Lauridsen et al. 2021). In addition, the transfer of vitamins and minerals from the sow to the piglet is limited from the prenatal stage, and the supply of some vitamins, which are important agents controlling stress effects, can become limiting at weaning (Matte and Audet 2020). Since weaning exposes the piglets to the above‐mentioned challenges, a vitamin–mineral supplementation via water could be an important strategy to increase hydration and to restore the animal's metabolism by providing essential nutrients to mitigate the negative weaning effects. The present study aimed to evaluate the impact of a flavored vitamin–mineral supplementation via water on the performance, oxidative status, and behavior of newly weaned piglets.

## 2 Material and Methods

All methods involving animal handling were performed in accordance with the regulations approved by the Institutional Animal Welfare and Ethics/Protection committee, Universidade Federal de Minas Gerais (UFMG), Brazil, under the protocol number 187/2019.

### 2.1 Animals and Experimental Procedure

This study was conducted in the nursery facilities of the swine production farm (NEPSUI) of the university UFMG, Brazil. A total of 90 piglets (45 females, 45 castrated males; TN70 × Talent) weaned at 24 days of age, with an initial body weight of 7.62 ± 1.28 kg, were used. The piglets were housed in pens of 1.20 m length × 1.00 m width × 0.80 m height, with a semiautomatic feeder, nipple drinker, and plastic slatted floor, with access to feed and water ad libitum. Three piglets were allocated per pen with no difference in the number of males and females between treatments. The piglets were allocated in a randomized block design, using initial body weight, sex, and litter origin as blocking factors, and were assigned to three treatments with 10 replicates per treatment. All animals received a complete diet formulated to meet or exceed the nutritional requirements for nursery pigs, including vitamin and mineral premixes, according to the recommendations of Rostagno et al. (2017). The treatments differed in the administration of an additional water‐soluble flavored vitamin–mineral supplement: a control group (CON), which received drinking water without any added supplement throughout the experimental period; a group supplemented with the vitamin–mineral solution via drinking water for 5 days postweaning and for an additional 5 days during the transition from Phase 3 to Phase 4, starting 3 days before the end of Phase 3 and continuing through the first 2 days of Phase 4 (W5D); and a group that received the same supplement continuously via water during the first 10 days after weaning (W10D). It is important to note that the water‐soluble supplement was offered in addition to the basal dietary vitamin levels already present in the feed and was not intended to replace dietary vitamin sources, but rather to serve as a complementary strategy to support piglet adaptation and oxidative balance during critical periods of postweaning stress. The flavored vitamin–mineral supplement (Eubiostart S, dsm‐firmenich, São Paulo, Brazil) used in the treatments was added to the drinking water at a concentration of 0.6 g/L. The supplement contained a composition of vitamins, per kg of product: A (7500 IU), 25OHD3 (2000 IU), D3 (3750 IU), E (62.50 IU), K3 (3750 mg), B1 (2500 mg), B2 (6250 mg), B6 (7750 mg), B12 (15,000 mcg), C (312.5 g), niacin (50 g), pantothenic acid (12.5 g), folic acid (1250 mg), biotin (187.5 mg), and mineral: Se (200 mg). The flavoring agent is a proprietary mixture of chemically defined aldehydes, ketones, and esters with sodium saccharin to impart a sweet fruit flavor to the water. Before the beginning of the trial, all nipple drinkers were individually calibrated to ensure uniform water delivery. Flow rate was measured using a 1‐L graduated cylinder and a chronometer, and drinkers were adjusted to provide a flow of 0.45 ± 0.05 L/min, which is within the recommended range for nursery pigs. In addition, the product was made available in the piglets' drinking water pipeline using a water‐powered doser pump (Dosmatic SuperDos 15TF, DosaMax, São Paulo, Brazil) tested for accuracy, and concentration checks were performed at the drinkers to confirm that the supplement was delivered at the intended 0.6 g/L across all pens, thereby avoiding any bias in water delivery among treatments. The nutritional program was divided into four phases: Phase 1 (24–28 days of age), Phase 2 (29–34 days of age), Phase 3 (35–49 days of age), and Phase 4 (50–67 days of age). The diets (Table 1) were formulated to meet the requirements of each category, following the recommendations of the Brazilian Poultry and Swine Tables (Rostagno et al. 2017).

**TABLE 1:** Composition of experimental diets (as to fed basis)^a^.

Ingredients	Phase 1	Phase 2	Phase 3	Phase 4
Corn	29.850	44.850	49.850	64.850
Soybean meal (CP 46%)	10.000	15.000	25.000	30.000
Precooked corn	21.937	11.026	8.626	—
Dried milk whey	18.000	12.000	6.250	—
Extruded soybean meal	4.500	4.000	2.500	—
Soybean protein concentrate	4.500	4.000	—	—
Plasma powder	4.500	2.000	—	—
Sugar	2.000	2.000	2.000	—
Inert	1.379	1.381	1.379	2.655
Limestone	—	—	—	0.760
Salt	0.250	0.400	0.400	0.400
Calcium sulfate	0.550	0.550	0.550	—
Dicalcium phosphate 19.5%	0.447	0.687	0.792	0.493
Benzoic acid	0.500	0.500	0.500	0.200
Zinc oxide 72% Zn	0.340	0.340	0.340	—
L‐lysine HCL	0.282	0.327	0.546	0.236
DL‐methionine	0.179	0.194	0.238	0.110
L‐threonine 98.5%	0.090	0.124	0.229	0.040
L‐tryptophan 98%	0.071	0.071	0.096	—
L‐valine	—	0.005	0.119	—
Antioxidant	0.265	0.185	0.225	0.000
Sweetener	0.125	0.125	0.125	0.020
Mineral premix	0.100	0.100	0.100	0.100
Vitamin premix	0.050	0.050	0.050	0.050
Carbohydrase	0.025	0.025	0.025	0.032
Protease	0.020	0.020	0.020	0.025
Amylase	0.020	0.020	0.020	0.014
Phytase	0.010	0.010	0.010	0.010
Xylanase	0.010	0.010	0.010	0.005
Calculated nutrient values				
Metabolizable energy, kcal/kg	3435	3412	3377	3367
CP, %	19.48	18.76	18.04	18.27
Calcium, %	0.683	0.710	0.715	0.720
Digestible phosphorus, %	0.439	0.430	0.400	0.360
SID lysine, %	1.35	1.30	1.26	1.20
SID methionine+ cystine, %	0.81	0.78	0.78	0.72
SID threonine, %	0.88	0.84	0.84	0.80
SID valine, %	0.95	0.91	0.88	0.84
SID tryptophan, %	0.30	0.29	0.25	0.23
Vitamin composition per kg of feed				
Vitamin A, IU	15,000	15,000	15,000	15,000
Vitamin D3, IU	3500	3500	3500	3500
Vitamin E, mg	200	200	200	100
Vitamin K3 (menadione), mg	4.5	4.5	4.5	4.5
Vitamin B1 (thiamine), mg	2.0	2.0	2.0	2.0
Vitamin B2 (riboflavin), mg	6.0	6.0	6.0	6.0
Vitamin B6 (pyridoxine), mg	3.5	3.5	3.5	3.5
Vitamin B12 (cyanocobalamin), mg	30	30	30	30
Niacin (nicotinic acid), mg	30	30	30	30
Pantothenic acid, mg	35	35	35	35
Folic acid, mg	4.0	4.0	4.0	4.0
Biotin, mg	1.0	1.0	1.0	1.0
Mineral composition per kg of feed				
Cu, mg	115	115	115	115
Fe, mg	160	160	160	160
I, mg	1.0	1.0	1.0	1.0
Mn, mg	40	40	40	40
Se, mg	0.4	0.4	0.4	0.4
Zn, mg	2588	2588	2588	2588
Cr, mg	0.20	0.20	0.20	0.20

*Note:* Mineral sources: carbo‐amino‐phospho chelates of copper (copper, Cu); carbo‐amino‐phospho chelates of chromium (chromium, Cr); carbo‐amino‐phospho chelates of iron (iron, Fe); carbo‐amino‐phospho chelates of manganese (manganese, Mn); carbo‐amino‐phospho chelates of selenium (selenium, Se); carbo‐amino‐phospho chelates of zinc (zinc, Zn); butyrate toluene hydroxide (BTH); calcium iodine (iodine, I).

Abbreviations: CP, crude protein; ME, metabolizable energy; SID, standardized ileal digestibility.

^a^ Phase 1 (24–28 days of age), Phase 2 (29–34 days of age), Phase 3 (35–49 days of age), and Phase 4 (50–67 days of age).

### 2.2 Measurements and Collected Data

Variations in ambient temperature, relative humidity, and photoperiod followed external conditions during the day. During the night, the rooms were protected with reinforced plastic curtains to block the wind and avoid exposing the animals to lower temperatures. Each pen was equipped with an individual heating infrared lamp, and all rooms had artificial illumination during the night. Ambient temperature and relative humidity were continuously recorded (1 measurement every 5 min) in the barns, using a data logger connected to a probe (Didai Tecnologia Ltda., Campinas, Brazil) placed 1 m above the floor. Every morning, feed refusals were collected when available, and fresh feed was immediately distributed once per day between 6:30 a.m. and 7:30 a.m. Feed intake was determined as the difference between the feed allowance and the refusals collected on the next morning. Feed samples were collected for further feed composition analyses.

The fecal score was evaluated twice a day: in the morning at 0800 h and in the afternoon at 1600 h, following the scoring proposed by Casey et al. (2007). The incidence of diarrhea followed a scale of 1–5, in which Score 1 represents dry and firm stools; Score 2 represents moist and soft stools, which are considered normal; Score 3 represents moist, soft, and pasty stools; Score 4 represents pasty diarrhea; and Score 5 represents watery diarrhea. The percentage incidence of diarrhea was calculated by dividing the number of diarrhea observations by the number of total observations and multiplying by 100. During the experiment, piglets that presented intense diarrhea (Scores 4–5) for more than 48 h were medicated with an intramuscular injection of 2.5‐mg dose of enrofloxacin during three consecutive days. Daily water consumption in each pen was also measured, always at the same time for each pen (i.e., between 1000 and 1100 h), using individual ultrasonic hydrometers (Sonata, Arad, Yoknem, Israel) installed in each pen.

Pigs were individually weighed at the beginning and at the end of each period of the experiment. For each growing stage, the average daily gain, average daily feed intake, and feed conversion were calculated. On Days 1, 10, and 28 of the experiment, at 0600 h, a saliva sample pool was collected from each pen via the use of a biting swab installed in the pens. The swabs with saliva were kept in 15‐mL Falcon tubes and immediately stored in a refrigerated cooler. The tubes were then centrifuged at 3000 rpm for 20 min at room temperature so that the saliva samples could be separated and sent for salivary cortisol determination. A competition ELISA test (Elabscience Laboratory—Porcine Cortisol ELISA Kit) was used to determine salivary cortisol. This ELISA test uses the “Competitive ELISA” principle in which the microplate of the ELISA kit is precoated with cortisol. Immediately after saliva collection, one piglet from each pen was randomly selected for blood sampling by puncturing the jugular vein into heparinized tubes, centrifuged at 3000 × g for 5 min at room temperature. After centrifugation, plasma and erythrocytes were separated, aliquoted, and stored at −80°C. Total superoxide dismutase (TSOD) and glutathione peroxidase (GPx) were analyzed in erythrocytes. Malondialdehyde (MDA) and vitamin C were analyzed in plasma. All biochemical analyses were realized at the Laboratory of Oxidative Stress (LEO)/UFRGS, Porto Alegre, RS, Brazil. The method described by Ōyanagui (1984) was used for the assay of TSOD activity using a TSOD assay kit A001 (Nanjing Jiancheng Bioengineering Institute). The method is because TSOD inhibits the generation of nitrite from oxidation of hydroxylamine by superoxide anion (O2^−^) that is produced by the xanthine/xanthine oxidase system. The activity of TSOD was expressed as units per milliliter and determined by measuring the reduction of optical density of the reaction solution at 550 nm with a spectrophotometer (UV‐ 2000, UNICO Instruments Co. Ltd., Shanghai, China). One unit of SOD was defined as the amount of SOD required to produce 50% inhibition of the rate of nitrite production at 37°C. The results were normalized for total protein content by the Bradford method, using bovine serum albumin as a standard.

GPx activity was determined using a GPx assay kit A005 (Nanjing Jiancheng Bioengineering Institute) (Maral et al. 1977). GPx is an enzyme that catalyzes glutathione oxidation by oxidizing the reduced tripeptide glutathione (GSH) into oxidized glutathione, considering that hydrogen peroxide is used as a substrate of glutathione. Absorbance was recorded at 412 nm. The GPx activity was expressed as units per mg of protein, and 1 unit was defined as a decrease in GSH of 1 mM/min after the decrease in GSH per minute of the nonenzymatic reaction was subtracted. The results were normalized for total protein (Bradford 1976).

MDA levels, an index of lipid peroxidation, were measured by high‐performance liquid chromatography (HPLC), employing a reversed‐phase column (SUPELCOSIL LC‐18‐DB HPLC column; 15 cm × 4.6 mm, 5 μm) and a mobile phase flow rate of 1 mL/min in 30 mmol/L monobasic potassium phosphate (pH 3.6) and methanol (9:1, v/v). The absorbance of the column effluent was monitored at 254 nm (Karatepe 2004). Under these conditions, the retention time of MDA was approximately 5 min. MDA levels were then calculated from an MDA standard curve.

Vitamin C (ascorbic acid) was measured by HPLC simultaneously with the MDA (Karatepe 2004). Under the same conditions, the retention time of vitamin C is approximately 3 min. Vitamin C levels were calculated from an ascorbic acid standard curve.

### 2.3 Behavioral Assessment

Behavior traits (feeding, drinking, and other activities) were monitored in the first 24 h after weaning, using a camera system installed in each one of the pens. One animal from each pen was randomly chosen for the behavioral assessment and marked with a colored stick so it could be easily identified. All recordings were analyzed using the EthoVision Observer XT software (Noldus, NL) in order to determine the differences in behavioral patterns between treatments (time to first water access, time to the first feed consumption, total time drinking water, total time eating feed, and frequency of visits to the drinker and feeder).

### 2.4 Calculations and Statistical Methods

The maximum, minimum, daily average, and variance of daily ambient temperatures and relative humidity were calculated and analyzed during the entire experimental period during the nursery phase. Data were submitted to normality test (Shapiro–Wilk) and analyzed using the mixed linear model (PROC MIX procedure) of the SAS statistical package (SAS Studio for academics), and differences were considered significant at *p* < 0.05 and tendency at *p* < 0.10. The effects of treatments and initial body weight were considered as a covariate effect and included in the statistical model. Analyses were conducted using the following statistical model:

*Y*_*ijk*_ = *μ* + *A*_*i*_ + *E* (*X*_*ijk*_ − *X*) + *e*_*ijkl*_, where *μ* = general average, *A*_*i*_ = treatments, *X*_*ijk*_ = observed value of the covariate “initial body weight”, *X* = average of the covariate “initial body weight,” *E* = regression coefficient between the covariate (*X*) and the response variable (*Y*), and *e*_*ijkl*_ = incidental residual effect of observation.

For the data that did not present normality (metabolic variables), to obtain the homogeneity of the variance, the adjustment of the data was realized by using the sinarc of the square root. According to the equation (Bolhuis et al. 2005)

Sinarc√*X* = *Y*

where

*X* = data collected;

*Y* = homogenized data.

The homogenized data were analyzed using a mixed linear model (PROC MIX procedure) of the SAS statistical package (SAS Studio for academics), and differences were considered significant at *p* < 0.05 and a tendency at *p* < 0.10. The ANOVA procedure in SAS (SAS Institute Inc., Cary, NC) was used for the behavioral analyses. Water and feed consumption behavior was analyzed using one animal from each pen as the experimental unit. Behavioral data were homogenized, converting nonparametric data into parametric data. For this, the square root of the arc sine was used, according to the equation proposed by Bolhuis et al. (2005). After homogenizing the variances, the data were then submitted to analysis of variance using the SNK test procedure of SAS. For the variable of incidence of diarrhea, the influence of each treatment on the occurrence of diarrhea was analyzed by applying the generalized linear model (GLM) in the SAS (1990) (GENMOD procedure).

## 3 Results

Average minimum and maximum temperatures and relative humidity levels measured during the experimental phase were 18.0°C and 35.9°C, and 32% and 89%, respectively. Piglet body weight at entrance did not differ (*p* = 0.582) among the treatments and averaged 7.62 ± 1.28 kg (Table 2). When compared with the CON and W5D, the DWG values for the W10D group were higher in Phase 2 (198 vs. 144 and 141 g/day, respectively, for W10D, CON, and W5D; *p* = 0.021) and Phase 3 (382 vs. 335 and 336 g/day, respectively, for W10D, CON, and W5D; *p* = 0.047; Table 2). As a result of an improved DWG, the treatments also influenced BW at the end of each phase, whereas pigs in the W10D treatment were significantly heavier at the end of Phase 2 (+600 g; *p* = 0.021), Phase 3 (+1197 g; *p* = 0.043), and at the end of the experiment (+1252 g; *p* = 0.002; Table 2) when compared with CON and W5D.

**TABLE 2:** Impact of flavored vitamin–mineral supplementation via water for piglets on overall nursery performance (LS means).

Parameters	Treatments			SEM^a^	*p* ^b^
	CON	W5D	W10D		
Phase 1 (1–4 day)					
Initial BW, kg	7.63	7.59	7.65	0.41	0.582
Final BW, kg	7.98	7.89	8.19	0.29	0.100
DFC, g/day	192	187	221	15	0.195
DWG, g/day	89	75	137	21	0.121
FC, g/g	2.16	2.49	1.61	0.56	0.339
DWE, L/day	1.99	1.97	2.03	0.21	0.987
Phase 2 (5–10 day)					
Final BW, kg	9.07b	8.99b	9.63a	0.33	0.021
DFC, g/day	265	257	305	15	0.112
DWG, g/day	144b	141b	198a	13	0.037
FC, g/g	1.84	1.82	1.54	0.23	0.130
DWE, L/day	1.91	2.02	2.46	0.16	0.151
Phase 3 (11–25 day)					
Final BW, kg	16.01b	15.99b	17.20a	0.50	0.043
DFC, g/day	513	502	556	17	0.585
DWG, g/day	335b	336b	382a	12	0.047
FC, g/g	1.539	1.495	1.457	0.02	0.335
DWE, L/day	2.91b	3.28b	4.06a	0.17	0.002
Phase 4 (26–42 day)					
Final BW, kg	27.93b	27.93b	29.19a	0.58	0.002
DFC, g/day	755	741	768	18	0.627
DWG, g/day	464	463	491	11	0.214
FC, g/g	1.634	1.594	1.563	0.02	0.243
DWE, L/day	4.19b	5.13a	5.54a	0.28	0.005

Abbreviations: BW, body weight; CON, control treatment, no supplementation; DFC, daily feed consumption; DWE, daily water expenditure; DWG, daily weight gain; FC, feed conversion; W10D, vitamin–mineral supplementation via water in the first 10 days after weaning; W5D, vitamin–mineral supplementation via water in the first 5 days after weaning and for another 5 days beginning at 3 days before the end of Phase 3 followed by the initial 2 days of Phase 4.

^a^ Standard error of the mean.

^b^ Within each phase, means with different lower‐case letters are statistically different (*p* < 0.05) or considered tendency (*p* < 0.10). Values represent the mean of 10 replicate pens per treatment (*n* = 10).

The treatments influenced water consumption throughout the experimental phase (Table 2). Although not significant during the first 10 days postweaning, the W10D treatment increased daily water intake by more than 0.498 L per piglet/day compared with CON and W5D. Treatments influenced significantly water intake for Phases 3 (*p* = 0.002) and 4 (*p* = 0.005), whereas W5D and W10D treatments increased piglet daily water consumption compared with CON (Phase 3: 4.069 vs. 3.280 vs. 2.912 L/day, respectively, for W10D, W5D, and CON; and Phase 4: 5.540 vs. 5.138 vs. 4.194 L/day, respectively, for W10D, W5D, and CON; Table 2).

Supplementation via water of the vitamin–mineral blend proved beneficial to the redox status of piglets during the postweaning period, reducing (*p* = 0.0189) the MDA plasma concentration on Day 10 in pigs from treatments W5D and W10D (1.28 vs. 1.39 vs. 3.94 μmol/mg, respectively, for W5D, W10D, and CON; Table 3). The supplementation of a vitamin–mineral complex via water tended to reduce plasma SOD concentration, with lower values in treatments W5D and W10D on Day 10 (*p* = 0.0726) and Day 28 (*p* = 0.0584) postweaning. During the experimental period, there were no significant differences (*p* > 0.10) in GPx activities between treatments (Table 3). The treatments did not differ statistically in terms of plasma vitamin C concentrations (*p* > 0.10; Table 3). The salivary cortisol level was also not influenced (*p* > 0.10) by the treatments throughout the entire study (Table 3).

**TABLE 3:** Impact of flavored vitamin–mineral supplementation via water for piglets on antioxidant metabolism parameters and salivary cortisol levels during the nursery stages (LS means).

Parameters	Treatments			SEM^a^	*p* ^b^
	CON	W5D	W10D		
SOD 1d, U/mg	16.46	17.49	16.12	0.90	0.8170
SOD 10d, U/mg	18.72	16.16	14.69	0.72	0.0726
SOD 28d, U/mg	16.17	13.20	13.18	0.59	0.0584
GPx 1d, U/g	9521	12,051	9188	892	0.3492
GPx 10d, U/g	15,116	16,390	16,711	1036	0.8439
GPx 28d, U/g	22,721	19,700	22,642	1703	0.7000
Vit. C 1d, μmol/mg	12.02	17.38	16.62	2.25	0.6122
Vit. C 10d, μmol/mg	13.58	29.07	19.83	4.64	0.3824
Vit. C 28d, μmol/mg	14.26	22.69	19.74	2.73	0.5088
MDA 1d, μmol/mg	3.23	1.36	3.71	0.53	0.1480
MDA 10d, μmol/mg	3.94b	1.28a	1.39a	0.43	0.0189
MDA 28d, μmol/mg	2.01	1.10	1.98	0.32	0.4828
Cort 1d, ng/ml	1.60	1.62	2.33	0.28	0.4104
Cort 10d, ng/ml	0.74	0.86	0.77	0.11	0.8573
Cort 28d, ng/ml	0.44	0.30	0.83	0.14	0.3284

*Note:* CON, control treatment, no supplementation; W5D, vitamin–mineral supplementation via water in the first 5 days after weaning and for another 5 days beginning at 3 days before the end of Phase 3 followed by the initial 2 days of Phase 4; W10D, vitamin–mineral supplementation via water in the first 10 days after weaning.

Abbreviations: 10d, 10th experimental day; 1d, 1st experimental day; 28d, 28th experimental day; Cort, salivary cortisol (ng/mL); GPx, glutathione peroxidase (U/g); MDA, malondialdehyde (μmol/mg protein); SOD, superoxide dismutase in erythrocytes (U/mg protein); Vit. C, vitamin C (μmol/mg protein).

^a^ Standard error of the mean.

^b^ Within each phase, means with different lower‐case letters are statistically different (*p* < 0.05) or considered tendency (*p* < 0.10). Values represent the mean of 10 replicate pens per treatment (*n* = 10).

The treatments showed a positive impact on the fecal score and incidence of diarrhea (Table 4). The pigs in treatments W10D and W5D had the lowest incidences of diarrhea compared with CON during the total nursery period (20.3 vs. 35.9 vs. 53.8%, respectively, for W10D, W5D, and CON; *p* < 0.001). However, considering the experimental phases, the W10D piglets showed the lowest diarrhea incidence in Phase 2 (30.8 vs. 49.9 vs. 64.2%, respectively, for W10D, W5D, and CON; *p* = 0.056), Phase 3 (1.6 vs. 25.0 vs. 38.3%, respectively, for W10D, W5D, and CON; *p* < 0.001), and Phase 4 (31.1 vs. 41.4 vs. 66.8%, respectively, for W10D, W5D, and CON; *p* = 0.002).

**TABLE 4:** Impact of flavored vitamin–mineral supplementation via water for piglets on the percentage of incidence of diarrhea during the nursery phases (LS means).

Phase	Treatments			SEM^a^	*p* ^b^
	CON	W5D	W10D		
Phase 1, %	35.2	25.2	20.2	4.1	0.350
Phase 2, %	64.2	49.9	30.8	5.3	0.056
Phase 3, %	38.3b	25.0b	1.6a	4.2	< 0.001
Phase 4, %	66.8b	41.4a	31.1a	5.2	< 0.002
Total period, %	53.8c	35.9b	20.3a	3.9	< 0.001

*Note:* CON, control treatment, no supplementation; W5D, vitamin–mineral supplementation via water in the first 5 days after weaning and for another 5 days beginning at 3 days before the end of Phase 3 followed by the initial 2 days of Phase 4; W10D, vitamin–mineral supplementation via water in the first 10 days after weaning. Phase 1: 1–4 days; Phase 2: 5–10 days; Phase 3: 11–25 days; Phase 4: 26–42 days; total period: 1–42 days.

^a^ Standard error of the mean.

^b^ Within each phase, means with different lower‐case letters are statistically different (*p* < 0.05) or considered tendency (*p* < 0.10). Values represent the mean of 10 replicate pens per treatment (*n* = 10).

The behavioral traits are presented in Table 5. During the first 24 h postweaning, the piglets in treatment W5D and W10D accessed the drinker faster than the CON piglets (01h05min vs. 01h10min vs. 02h42min, respectively, for W10D, W5D, and CON) and showed less time to first contact with the feed trough and starting eating faster compared with CON (02h08min vs. 02h35min vs. 08h20min, respectively, for W5D, W10D, and CON). Although there was no statistical difference (*p* = 0.336), still W10D piglets spent a higher percentage of time at the drinker compared with W5D and CON (0.46 vs. 035 vs. 0.31%, respectively, for W10D, W5D, and CON). Piglets from the W10D and W5D treatments showed a higher time spent eating compared with CON, and when compared among both water treatments, W10D showed an increase compared with W5D (6.91 vs. 4.03 vs. 2.82%, respectively, for W10D, W5D, and CON; *p* = 0.045). In contrast, CON pigs showed a higher time spent exploring the pen when compared with W10D and W5D pigs (20.23 vs. 8.31 vs. 8.32%, respectively, for CON, W5D, and W10D; *p* = 0.032). Time spent sleeping (on average 54%) and inactive (on average 27%) was not influenced by the treatments.

**TABLE 5:** Impact of flavored vitamin–mineral supplementation via water for piglets on the behavioral traits during the first 24 h postweaning.

Parameters	Treatments			SEM^a^	*p* ^i^
	CON	W5D	W10D		
Time 1st FI (h)^b^	08h20min	02h08min	02h35min	—	—
Time 1st WI (h)^c^	02h42min	01h10min	01h05min	—	—
TDrink (%)^d^	0.31	0.35	0.46	0.82	0.336
Teat (%)^e^	2.82b	4.03b	6.91a	1.57	0.045
TExploring (%)^f^	20.23b	8.31a	8.32a	1.89	0.032
TInactive (%)^g^	23.02	32.31	28.31	3.46	0.956
TSleeping (%)^h^	53.62	55.00	56.00	2.61	0.965

*Note:* CON, control treatment, no supplementation; W5D, vitamin–mineral supplementation via water in the first 5 days after weaning and for another 5 days during the transition from starter diet I to starter diet II; W10D, vitamin–mineral supplementation via water in the first 10 days after weaning.

^a^ Standard error of the mean.

^b^ Hours to 1st ingestion.

^c^ Hours to 1st water intake.

^d^ Time spent on drinker.

^e^ Time spent on feeder.

^f^ Time spent exploring the pen.

^g^ Time spent inactive.

^h^ Time spent sleeping.

^i^ Within each phase, means with different lower‐case letters are statistically different (*p* < 0.05) or considered tendency (*p* < 0.10). Values represent the mean of 10 individual piglets per treatment (*n* = 10).

## 4 Discussion

Weaning is a challenging moment for the piglet and is associated with adverse effects during this stage: the separation from the sow, environmental changes (shifted to other barns), resocialization with other litters, and an abrupt change of the dietary profile, whereas dietary stress is a result of replacing easily digestible sow milk with mainly plant‐based solid feed. All these produce negative stress in the piglet and a neophobia to the feed that results in a low feed intake at the weaning (da Silva et al. 2020).

Few studies have evaluated the voluntary water intake of weaned piglets. However, existing data indicate that both water and feed consumption tend to be low during the first 48 h after weaning (da Silva et al. 2020; de Sousa Luna et al. 2023). The use of flavoring agents in water may improve palatability and encourage drinking behavior, thus contributing to hydration and overall adaptation during this critical phase. Our findings agree with this statement, where piglets supplemented with the flavored (sodium saccharin) vitamin–mineral mixture ingested more water, especially during the first 10 days after weaning, a 24% increase in water intake during nursery. According to da Silva et al. (2020), the improvement in piglets' weight gain can be a direct influence of hydration and nutrient intake on intestinal villi length development and an increase in the crypt's depth, improving feed efficiency usage. Our findings agree with the statement, whereas piglets from the W10D showed a higher water intake, increased daily weight gain (+16%) and end weight (+6%) on Day 10 postweaning and a higher BW at the end of nursery (+5%) when compared with CON and W5D.

In the weaning phase, piglets often show growth depression and are more susceptible to diseases, a phenomenon known as postweaning stress syndrome (Pluske et al. 1997). They are also more vulnerable to free radicals as their antioxidant system is unprepared (Xu et al. 2014). Cao et al. (2018) showed that the first week of nursery is characterized by an increase in the MDA concentration. Similarly, in the present study, on Day 10 postweaning, the MDA values of the control animals were three times higher when compared with the animals supplemented with the vitamin–mineral complex. The positive effects on the redox status of weaned piglets were due to the inclusion of Selenium and important antioxidant vitamins in the water, such as A, E, and C. It is important to clarify that all piglets received a nutritionally complete diet with vitamin and mineral levels formulated according to Rostagno et al. (2017), which in several cases exceed the recommendations of NRC (2012) and the levels commonly used in international studies. Thus, the water‐based supplementation was not intended to replace dietary sources but to provide a functional antioxidant boost during the postweaning period. For example, although the amounts of vitamins A and E provided via water (4.5 and 0.0375 IU/L, respectively) were indeed negligible compared with the basal diet (15,000 and 200 mg/kg), these values still exceeded the levels reported in some recent studies, such as Choi et al. (2024), who included 6614 IU/kg of vitamin A and 19.8 IU/kg (approximately 13.2 mg/kg) of vitamin E in the basal diet. Therefore, the improved oxidative status observed in the supplemented groups may be more reasonably attributed to the additional intake of B‐complex vitamins, vitamin C, K, and selenium, which were present in the supplement at functionally effective concentrations. In agreement with this study, findings from other authors have reported that weaned piglets supplemented with Se (Liu et al. 2021); vitamin A (Zhou et al. 2021) or its precursors, as β‐carotene (Li et al. 2020), showed a reduction in MDA concentration and a more balanced redox status.

Although increased water intake can positively influence early‐postweaning adaptation (da Silva et al. 2020), hydration alone does not explain the reductions in MDA and SOD observed in the supplemented groups. Studies evaluating flavored water without added vitamins or minerals have reported increases in water consumption and feed intake but did not show improvements in oxidative‐stress biomarkers (da Silva et al. 2020; de Sousa Luna et al. 2023). Therefore, the antioxidant effects detected in the present study are more consistent with the known biochemical functions of the vitamins and selenium present in the supplement, rather than with increased water intake in isolation.

Similarly, according to the observations of Wu et al. (2023), vitamin D can also attenuate oxidative stress by restoring GPx activity through binding to vitamin D receptors (VDR) and reducing MDA concentration. In addition, it was reported in another study that preslaughter supplementation of pigs with vitamin D3 promoted a reduction in MDA and a trend towards a reduction in serum cortisol (Rey et al. 2020). However, in the present study, only a reduction in the plasma concentration of MDA was observed. Meanwhile, the antioxidant effect of vitamin K is not widely described in pigs, but rather in studies with rodents used as an experimental model (Lai et al. 2022). However, in agreement with this study, vitamin K is also associated with the reduction of MDA in the body. It is described as a vitamin with an important role in the maintenance of the antioxidant system and in the activity of antioxidant enzymes (Lai et al. 2022). Similarly, vitamin B complex is also not very well described in research with pigs, but the participation of these vitamins has been reported in studies with birds (Miladinović et al. 2021). In this case, for quails subjected to environmental challenges, a reduction in MDA concentration and an increase in SOD activity were observed in the groups supplemented with vitamin B. Although piglets were not exposed to additional environmental challenges during the trial, the stressors inherent to weaning (separation from the sow, dietary changes, and alterations in the living environment) were sufficient to compromise performance and health in the control group. This was evidenced by increased incidence of diarrhea and reduced growth rate in the control group. Therefore, the observed improvement in redox status in the supplemented groups suggests that the vitamin–mineral additive helped to mitigate the oxidative impact of weaning‐related stressors.

Increased production of ROS, particularly superoxide anions, is known to stimulate the upregulation of antioxidant enzymes such as SOD as a compensatory cellular response to oxidative challenge (Cao et al. 2018; Degroote et al. 2020; Novais et al. 2020). Because SOD activity increases in response to higher superoxide production, a reduced oxidative challenge would diminish the stimulus for SOD upregulation. Therefore, the simultaneous decrease in both biomarkers is consistent with a scenario of lower ROS formation.

Vitamin E is the main exogenous antioxidant agent, effective in protecting the cell membrane against the harmful effects of oxidative stress, neutralizing free radicals (Buckley et al. 1995). According to Amazan et al. (2012), supplementation of α‐tocopherol via water was responsible for increasing the concentration of antioxidant enzymes in sows and piglets. Along the same line, ascorbic acid is an important antioxidant and detoxifying agent for weaned piglets (Su et al. 2018). Shi et al. (2017) supplementing piglets with vitamin C showed a higher concentration of antioxidant enzymes in these animals. In our study, we did not observe a significant difference in vitamin C levels and in the expression of the antioxidant enzyme GPx, but we did see a tendency for lower SOD levels on Days 10 and 28 for the piglets that received the supplement. Superoxide dismutase helps break down potentially harmful oxygen molecules in cells and is highly sensitive to any slight change in the redox state of the cell, and its level rises in response to the elevated cellular oxidative stress. Therefore, one can hypothesize that the lower levels of SOD observed in our study may result from a reduced stimulation of the antioxidant system by ROS. The SOD activity and the lower concentration of MDA observed in piglets treated with W5D and W10D may indicate that these animals were able to maintain their metabolic homeostasis with the support of additional vitamin–mineral supplementation. In this context, with a lower concentration of MDA, there was also a reduced need to produce antioxidant agents. However, to support our hypothesis, future research is still needed, as there are no published studies to our knowledge that have validated this association. As for the vitamin C findings in our study, it is important to note that pigs can synthesize ascorbic acid endogenously, mainly in the liver, and therefore, vitamin C is not considered an essential dietary nutrient under normal physiological conditions (NRC 2012). However, the postweaning period is characterized by intense metabolic, oxidative, and psychological stress, which can temporarily exceed the piglets' endogenous capacity for vitamin C synthesis.

According to Deng et al. (2020), the effect of oxidative stress is detrimental to intestinal integrity. Thus, the impairment of intestinal structures can interfere with the basic absorption functions and also barrier protection, leaving the piglet more vulnerable to infections and the occurrence of diarrhea (Heo et al. 2013). After weaning, chronic social stress is also responsible for physiological imbalance, increased membrane permeability, intestinal immunosuppression, and the establishment of a more proinflammatory status. This breakdown in homeostasis is responsible for the impact on performance indexes and worse feed conversion (Li et al. 2020). Similar to other studies, which evaluated the use of vitamin B6 (Li et al. 2019) and vitamin A (Wang et al. 2020) on the incidence of diarrhea in piglets during the postweaning period, in the present study, the W10D group animals showed a reduction in the incidence of diarrhea throughout the entire nursery phase.

Weaning is characterized as a resocialization period for the piglets, which alters the social behavior of the newly formed group. Thus, aggression between animals of different litters is commonly observed and causes social stress (Merlot et al. 2004; Coutellier et al. 2007). The chronic social stress caused by weaning can be measured by an increase in salivary cortisol concentration, which leads to the triggering of pathophysiologies and, consequently, negative impacts on performance (Li et al. 2020). The absence of differences in salivary cortisol levels among treatments may be attributed to the limited group size (three piglets per pen) and similar body weight range, which potentially minimized aggressive interactions. Nevertheless, the observed improvements in antioxidant parameters and reductions in diarrhea and weight loss in the supplemented groups suggest that the weaning process still imposed sufficient physiological and metabolic stress to trigger measurable oxidative responses, even in the absence of overt social conflict.

In conclusion, it might be safely deducted from our study that the use of a flavored vitamin–mineral supplement in the drinking water of the piglets postweaning has the potential to improve piglet performance. Our findings lead us to believe that the strategic use of this nutritional technology via water to weaned piglets for 10 consecutive days is a viable strategy to increase the piglet's voluntary water intake, with positive effects on the redox status of the animals, as well as the reduction of diarrhea incidence and performance improvements throughout the nursery period, all of which can protect the animal from the negative effects of the weaning process.

## Funding

The authors have nothing to report.

## Conflicts of Interest

The authors declare that the main role of dsm‐firmenich in this study was through the donation of the tested additive and to support financially the serum laboratorial analyses.
